# Pediatric postural orthostatic tachycardia syndrome: From mechanisms to individualized management

**DOI:** 10.1002/pdi3.2509

**Published:** 2024-11-24

**Authors:** Wenrui Xu, Hongfang Jin, Ying Liao, Junbao Du

**Affiliations:** ^1^ Department of Pediatrics, Children's Medical Center Peking University First Hospital Beijing China

**Keywords:** mechanism, postural orthostatic tachycardia syndrome (POTS), treatment

## Abstract

Postural orthostatic tachycardia syndrome (POTS) represents chronic orthostatic intolerance. Patients usually suffer from presyncopal symptoms, such as lightheadedness, headache, blurred vision, and fatigue. Central hypovolemia, peripheral vascular dysfunction, and a hyperadrenergic state may contribute to the pathogenesis of POTS. It is necessary to comprehensively assess the clinical characteristics, physiological changes, and biochemical markers to select an appropriate therapy. Since the introduction of the concept of individualized treatment for POTS, great progress has been made in the field.

## 1 INTRODUCTION

Syncope is defined as the transient loss of consciousness with decreased muscle tone and collapse caused by cerebral hypoperfusion. Syncope accounts for approximately 1% of all emergency room visits.^1^ Neurally mediated syncope accounts for 70%–80% of pediatric syncope cases, and postural orthostatic tachycardia syndrome (POTS) is one of the most common diseases.^2^, ^3^ POTS represents chronic orthostatic intolerance. Patients usually suffer from presyncopal symptoms, such as lightheadedness, headache, blurred vision, and fatigue. Some of them may faint. The symptoms mostly occur when the position changes from supine to upright but can also appear in any posture. Hemodynamic changes are characterized by an excessive increase in heart rate (HR) in the absence of hypotension that occurs within the first 10 min of head‐up.^4^

In children with POTS, the primary characteristic feature is sustained, excessive tachycardia in the non‐recumbent position. Central hypovolemia, peripheral vascular dysfunction, and a hyperadrenergic state may contribute to the pathogenesis of POTS.^5^ Based on these factors, treatments for POTS mainly aim to increase central volume, improve vasodilation status, antagonize disordered neurohormones, and regulate imbalanced autonomic function. The effectiveness of each treatment has been reported previously.^6^ Therefore, it is necessary to comprehensively assess the clinical characteristics, physiological changes, and biochemical markers to select an appropriate therapy. Since the introduction of the concept of individualized treatment for POTS, great progress has been made in the field.

## 2 THE CURRENT STATUS OF INDIVIDUALIZED TREATMENT OF POTS IN CHILDREN

The management of POTS includes both non‐pharmacological and pharmacological interventions. Therefore, patient education is fundamental.

### 2.1 Physical treatment in treating children with POTS

Cardiovascular deconditioning significantly contributes to POTS and related functional disability. Exercise training and physical treatment such as orthostatic training, horizontal exercise and physical countermeasure maneuvers that could improve autonomic function are recommended.^7^ However, not all patients respond positively to these physical treatments.

Lu et al. found that children with POTS had longer HR‐corrected QTd (QTcd) than healthy children, and that QTcd in responders to physical training was longer than that in non‐responders. The area under curve (AUC) of the receiver operating characteristic (ROC) curve, which was used to evaluate the response prediction power, was 0.73. Using 43.0 ms as a cutoff value yielded a high sensitivity of 90% in predicting an effective response, whereas the specificity was only 60%, which was not satisfactory.^8^

### 2.2 Oral rehydration salts in treating children with POTS

Some pediatric patients with POTS have insufficient water and salt intake which results in an inadequate circulating capacity. Oral rehydration salts (ORS) is an acceptable therapeutic option for correcting relative hypovolemia. However, its effectiveness varies among children with POTS due to the inconsistent presence of low blood volume and reduced sodium loading.

Recently, researchers found that the baroreflex sensitivity (BRS) value could be a biomarker for predicting the response to ORS in children with POTS, demonstrating both high sensitivity and specificity.^9^ However, acquiring a BRS requires unique equipment, limiting its application. There are other indices more convenient to obtain, that have also been useful in predicting the therapeutic effectiveness of ORS, such as the mean corpuscular hemoglobin concentration (MCHC) and changes in HR during head‐up tilt test (HUTT).^10^, ^11^ However, both indices had unsatisfactory specificity and sensitivity. Additionally, obtaining MCHC involves drawing blood, which may cause pain and discomfort.

The excretion of 24‐h urinary sodium level reflects sodium loading and, therefore, could indicate the central volume status.^12^ Zhang et al. found that basal 24‐h urinary sodium levels were lower in children diagnosed with POTS than in healthy controls. A 24‐h sodium excretion of less than 124 mmol/24 h indicated the effectiveness of ORS in treating pediatric POTS, with a sensitivity of 76.9% and specificity of 93.0%. The ROC curve showed an AUC of 0.879.^3^, ^13^ BMI can reflect blood volume to some extent. Li et al. found that patients with POTS who responded to ORS had a low BMI. A cutoff value of 18.02 kg/m^2^ yielded a sensitivity of 92.0% and specificity of 82.8% in predicting the therapeutic response; the ROC curve revealed an AUC of 0.923.^14^

As mentioned above, BMI as a marker to predict the reponders to ORS in POTS patients had a large AUC in ROC analysis. Furthermore, it is more convenient and economical than 24‐h urine sodium measurement. Therefore, predicting the efficacy of ORS therapy in children with POTS is recommended. However, it should be noted that BMI may be affected by age, sex, and body fat content during childhood, which merits further investigation to increase its predictive value.

### 2.3 *β*‐Adrenergic receptor blockers in treating children with POTS

Tachycardia associated with the orthostatic position is a clinical feature of POTS. *β*‐Adrenergic receptor blockers can block the effect of excessively increased catecholamine levels in some patients to alleviate symptoms. Biomarkers or indices reflecting hyperadrenergic status could guide the therapeutic response to *β*‐adrenoreceptor blockers, such as metoprolol.

Zhang et al. found that children with POTS and higher orthostatic plasma norepinephrine (NE) showed a better response to metoprolol, with an AUC of 0.785. Taking 3.59 pg/mL as the boundary value yielded a sensitivity of 76.9% and specificity of 91.7%.^15^ Similarly, the basal plasma C‐type natriuretic peptide level in responders to metoprolol was significantly higher than that in non‐responders, whereas the basal plasma copeptin level was lower in responders.^16^, ^17^ All of these biomarkers could be used to predict clinical improvement related to metoprolol treatment in pediatric POTS. However, these measurements are acquired using invasive methods. Additionally, orthostatic plasma NE requires blood sampling in an upright position, which is more complex.

Wang et al. evaluated the usefulness of HR difference during HUTT to predict treatment outcomes. They found that patients with a higher HR change between 5 and 0 min showed a better response to metoprolol. An HR increase by 34 beats/min could be used as the cutoff value, with a sensitivity of 85.29% and a specificity of 89.47%.^18^ Although HUTT is a non‐invasive examination method, it may reproduce the patient's symptoms and cause discomfort, even evoking syncope.

To explore a more precise and accessible method, Xu et al. developed an electrocardiogram‐based model to predict the effectiveness of metoprolol therapy in children with POTS. Three indices, including the maximum value of the P‐wave after correction (Pcmax), minimum value of the QT interval after correction (QTcmin), and T‐peak‐to‐T‐end interval dispersion (Tped), were combined. The model yielded high predictive sensitivity (93.8%) and specificity (90.0%), with an AUC of 0.970.^19^ While promising, simpler methods are needed for wider application.

### 2.4 *α*‐Adrenergic receptor agonists in treating children with POTS

Some patients with POTS have peripheral vascular dysfunction, primarily due to peripheral neuropathy, leading to what is termed neuropathic POTS. In these patients, peripheral vessels can be excessively dilated or insufficiently contracted due to increased blood flow in certain vessel beds, especially when affected by gravity. Hence, patients with POTS exhibit abnormally excessive vasodilatation might benefit from *α*‐adrenoreceptor agonists like midodrine hydrochloride. Biomarkers indicating vascular dysfunction could aid in identifying potential responders.

Adrenomedullin (ADM) is an unstable vasodilator with a short half‐life. In contrast, midregional pro‐adrenomedullin (MR‐proADM) remains stable in plasma and can serve as an indicator of ADM levels. Research showed that children who responded well to midodrine hydrochloride had elevated plasma MR‐proADM levels. With an AUC of 0.879, using a cutoff value of 61.5 pg/mL achieved a sensitivity of 100% and specificity of 71.6%.^20^ Additionally, plasma copeptin level >10.48 pmol/L and erythrocytic hydrogen sulfide production rates >27.1 nmol/min/10^8^ erythrocytes were found to predict the efficacy of midodrine hydrochloride in children with POTS.^21^, ^22^ However, assessing these indices necessitates blood sampling. Seeking a noninvasive alternative, Deng et al. examined hemodynamic parameters during the standing test, suggesting that increased blood pressure during this test might predict treatment outcomes.^23^ Although the standing test is cost‐effective and routine for POTS diagnosis, its repeatability raises concerns. More clinical studies are essential to validate its predictive value. Liao et al. noted that children with elevated basal flow‐mediated dilatation (FMD) responded positively to midodrine hydrochloride. The predictive value of FMD, as indicated by the ROC curve, was 0.790. With a threshold of 9.85%, sensitivity and specificity stood at 74.4% and 80.0%.^24^ Despite the high AUC of plasma MR‐proADM, FMD is favored due to its non‐invasive and stable characteristics.

## 3 SUMMARY AND PERSPECTIVE

Individualized treatment of children with POTS is an ideal therapeutic strategy. For patients, identifying the primary pathogenic mechanism and then implementing targeted therapy can effectively improve the treatment response. Studies have shown that therapeutic effects can be predicted using several biomarkers or indicators. However, most studies focused on a single index, and the present results were mainly obtained from small sample sizes. High‐quality, multicenter, large‐scale clinical studies are still lacking to verify the effectiveness of these indicators. Additionally, the pathogenesis of POTS is not fully understood, and mechanisms other than those mentioned in this review, such as autoimmune disorders, deconditioning, and mast cell activation, may be involved. Further investigations combining basic research with clinical manifestations are needed to better understand the nature of the disease. Therefore, introducing new biomarkers to guide future therapies and integrating a network of indicators for therapeutic effect prediction can establish comprehensive prediction models, enhancing efficiency.

## AUTHOR CONTRIBUTIONS

**Wenrui Xu**: Methodology; writing—original draft. **Junbao Du**: Conceptualization; funding acquisition; writing—review and editing. **Hongfang Jin**: Conceptualization; supervision; writing—review and editing. **Ying Liao**: Methodology; writing—review and editing.

## CONFLICT OF INTEREST STATEMENT

The authors declare no conflicts of interest.

## ETHICS STATEMENT

Not applicable.

## Data Availability

The data that support the findings of this study are available from the corresponding author upon reasonable request.
